# Reimagining the frailty review: meaning, metrics, and the missed opportunity in global ageing care

**DOI:** 10.3389/fmed.2025.1620193

**Published:** 2025-11-05

**Authors:** Waseem Jerjes, Daniel Harding, Arnoupe Jhass

**Affiliations:** ^1^Hammersmith and Fulham Primary Care Network, London, United Kingdom; ^2^Faculty of Medicine, Imperial College London, London, United Kingdom

**Keywords:** frailty review, person-centered care, falls, polypharmacy, hospitalization, mortality, physical function, cognition

## Introduction

Frailty—across physical, cognitive, psychological, and social domains—affects more than 10% of older adults in England, with prevalence rising steeply with age (1), and similar rates are reported internationally, with associated risks of falls, hospitalization, and mortality (2–4). The UK's National Health Service has embedded frailty into policy, such as the Aging Well programme, anticipatory care planning, and the EHCH model (1). Similar frameworks—such as Australia's My Aged Care system and Canada's Home First approach—also seek to embed frailty planning in primary and community care (5, 6). Within primary care, the implementation of the yearly frailty review has been framed as the cornerstone of this strategy—intended to enable proactive, personalized care for older adults with multiple health and social issues. Because physical performance is modifiable, the frailty review should routinely initiate or adjust an individualized exercise plan—particularly progressive resistance and balance training—linked to patient-defined goals.

In this paper, a frailty review refers to a scheduled, structured primary-care consultation that brings together frailty identification, goal-setting, medication review, and anticipatory care planning for older adults. In NHS England, this is an established annual review, but analogous review points exist internationally within primary and community care. Here, we treat frailty as a multidimensional construct spanning physical function, cognition, psychological wellbeing, and social support; unless specified, references to “frailty” and “frailty review” encompass all four domains.

But for most patients, reviews like this are far from personal. Audits show that frailty reviews are all too often formulaic, risk-led (e.g., falls, polypharmacy, and hospitalization), and more decided by clinical proformas than by meaningful conversation (7). While the “what matters to me” words have been used across the board, patients are unclear about the purpose of the review and think their values, goals, and daily realities remain hidden (8, 9).

This opinion paper asserts that the manner in which frailty reviews are being rolled out—albeit with good intentions—risks overlooking the very essence of person-centered care. From the patient perspective, it looks at the disparity between what policy statements commit to and what patients actually receive. According to the best available evidence, it challenges us not just to ask whether frailty reviews are being done, but whether they are addressing the expectations, dignity, and voice of the people they are attempting to support—regardless of country or system.

### Frailty reviews: global aspirations vs. lived realities

Across several health systems, structured frailty reviews have emerged as key tools for proactive care. In the UK, the annual frailty review was intended as a mid-year pause point: a time for mutual reflection, goal setting, and preventive planning for clinician and patient. Its enshrinement in primary care contracts puts it as a formalized opportunity for the individualization of care for those most likely to deteriorate. Ideally, it would shift the focus from a crisis-driven response to anticipatory, relationship-based care that responds to the patient's own agenda (10).

However, patient reports indicate that the intended benefits are frequently not realized in practice. In Canada, for instance, a national evaluation of home-based frailty reviews highlighted similar patient dissatisfaction with impersonal assessments and lack of follow-up (11). Similarly, recent efforts in China to develop psychological frailty indices have revealed the challenge of connecting metric-driven models with lived experience (3). Time pressure and target-driven templates often convert the review into a checklist consultation, which narrows the space for goals and follow-up. The review can function primarily as an administrative exercise rather than a relational encounter.

In one recent national evaluation of frailty management in primary care, patients reported that reviews were felt as surveillance, rather than support, when provided by unfamiliar clinicians or with a lack of follow-up (12). For others, the experience was not only impersonal but also disorienting: the frailty reviews marked a shift in the manner in which they understood themselves—not as persons with priorities but as problems to be fixed (13). A qualitative metasynthesis of patient experiences described the reviews as “functionally efficient but emotionally absent,” capturing the wider tension between system-level measurement and personal meaning (14).

This expanding gap between design and delivery requires urgent action. If frailty reviews must become more than annual exercises, then they must be redesigned as moments of therapeutic opportunity—moments where the patient's voice is not just invited but placed centrally, and where continuity, curiosity, and compassion characterize the clinical interaction. Overall, delivery remains template-led and target-driven, with limited continuity and limited follow-up.

### When “what matters” becomes a checkbox

Few phrases have gained such widespread currency in contemporary frailty care as “what matters to me.” It appears across care guidelines in the UK, the US, and the Netherlands as a signal of relational, value-led working (1, 15, 16), personalized care systems, and dozens of quality improvement initiatives as shorthand for relational, value-led working. As with much good intent, its operationalization risks descending into performance over substance.

In the majority of practices, “what matters to me” appears as a question in the frailty review template—often near the end of a structured form. This has the effect of subverting the priorities: rather than being the focus of the discussion, the patient's priorities appear to be an afterthought, added on to pre-gathered clinical information. Some clinicians, rushed for time or uncertain how to respond, put down vague or minimal answers: “remain independent,” “avoid hospitalization,” or “no issues raised.” In these circumstances, the intent of the question is preserved, but its influence on decisions is limited.

Patients also differentiate between being heard and being asked. A recent review on the exploration of personalized care planning in older adults found that while the majority of participants were formally invited to express preferences, few thought that their responses were influential in decisions that were then taken (17). For some, the question was emotionally charged, eliciting considerations of loss, purpose, or dependency—but there was not much time and space to explore these lines of thought before the review continued.

When questions about what matters are recorded as data points rather than being used to organize decisions, they do not shape the plan. Personalization follows when the answer to what matters orders the subsequent steps. In practice, personalization is most evident when the plan reflects the answer to what matters.

### What we miss when we focus only on risk?

Risk terms such as falls, delirium, and hospitalization describe only part of frailty. Small functional and social losses reshape daily life to an equal degree. For most patients, frailty is not the dramatic change but the accumulation of incremental, unseen losses: not kneeling in the garden, not going down the stairs after dusk, not being able to make it to the family gathering for fear of incontinence. These are not typically charted, but they remake daily life as much as any measurement.

Current frailty reviews, with all the focus on risk stratification, hardly provide space for such quiet stories. Even when patients are questioned about what matters, the general format of the review pulls the discussion back to measurable outcomes—mobility, nutrition, and medication. Emotional aspects of aging—such as sorrow, anxiety, and frustration—are only partially addressed. In Taiwan, Brazil, and many European countries, emotional resilience is increasingly being integrated into frailty models, with frameworks recognizing that psychological loss can precede functional decline (18).

This exclusion is not significant. Emotional health has been linked with functional decline, compliance, and health-seeking behavior in older adults (3, 19). The system, however, acts as though emotional responses to frailty are inevitable or immutable, rather than as expressions of unmet need. Without an open invitation, patients do not share such experiences—particularly if they are afraid of being seen as burdensome or negative.

Continuity of care plays the essential function of spanning this gap. Individuals who retain the same clinician over a period of time are more inclined to discuss emotional or existential concerns and to be listened to as an individual rather than receive a diagnosis. Systematic review has for many years demonstrated that continuity with a known doctor is associated with lower mortality, increased satisfaction, and increased uptake of preventive care (20). In the context of frailty, such continuity is not a luxury—it is the interpersonal glue that makes it possible to discuss loss and purpose.

Non-clinical goals take the central place of what really counts: making one's own tea, getting to the corner shop, and attending the wedding of a granddaughter. These are not unimportant goals. They are about identity, relationships, and independence. When a review of frailty omits them, it not only omits what clinically has to take priority but also what gives life its worth. Routine review that attends to functional, social, and emotional losses alongside risk metrics offers a more accurate account of the lived experience.

### Patient agency and the hidden power of being asked

In frailty, where the course of health appears predetermined, the reestablishment of agency can be both preventive and therapeutic. For older persons, the shift from independence to dependence can be insidious, gradual, and very personal.

Simply asking what is important has the power to disrupt that dynamic. It repositions the patient not as a person who simply waits for care, but as a person whose preferences and priorities are important. Even where the answer cannot alter the clinical course, the act of asking conveys the message of attention, respect, and acknowledgment (21).

There is mounting evidence that goal-setting, particularly when rooted in the patient's context and words, enhances engagement, satisfaction, and follow-through. Meta-analysis of personalized care planning showed that older adults who were actively involved in the process of setting goals for themselves were more confident in being able to control health and were more likely to utilize support services (22). Importantly, the most meaningful goals were often humble—getting back to the usual hobbies, joining a social group, and walking unaided in the home.

When taken seriously, the objectives achieve a shift in how the patient is connected to the system. Care is delivered collaboratively rather than unilaterally. So, asking what is important is not courtesy—it is therapeutic intervention in itself, one that builds autonomy, trust, and a therapeutic relationship. It re-establishes something easily lost in long-term care: the sense that one's life story is still being written, not just recorded. When goals are elicited and acted upon, patients' sense of agency and engagement increases.

### Redesigning the review around meaning, not metrics

The frailty review was intended to be the linchpin of personalized care—a formalized opportunity to stand still, take stock, and plan ahead with the older person as the central figure. To realize that vision, however, it must be more than a decent-intentioned appointment. It should operate as a relational process that integrates the patient's narrative with clinical data. Redesigning the review requires not merely practical changes in delivery but also a deeper shift in the way the healthcare system conceives of time, meaning, and care in later life.

**Figure 1:**
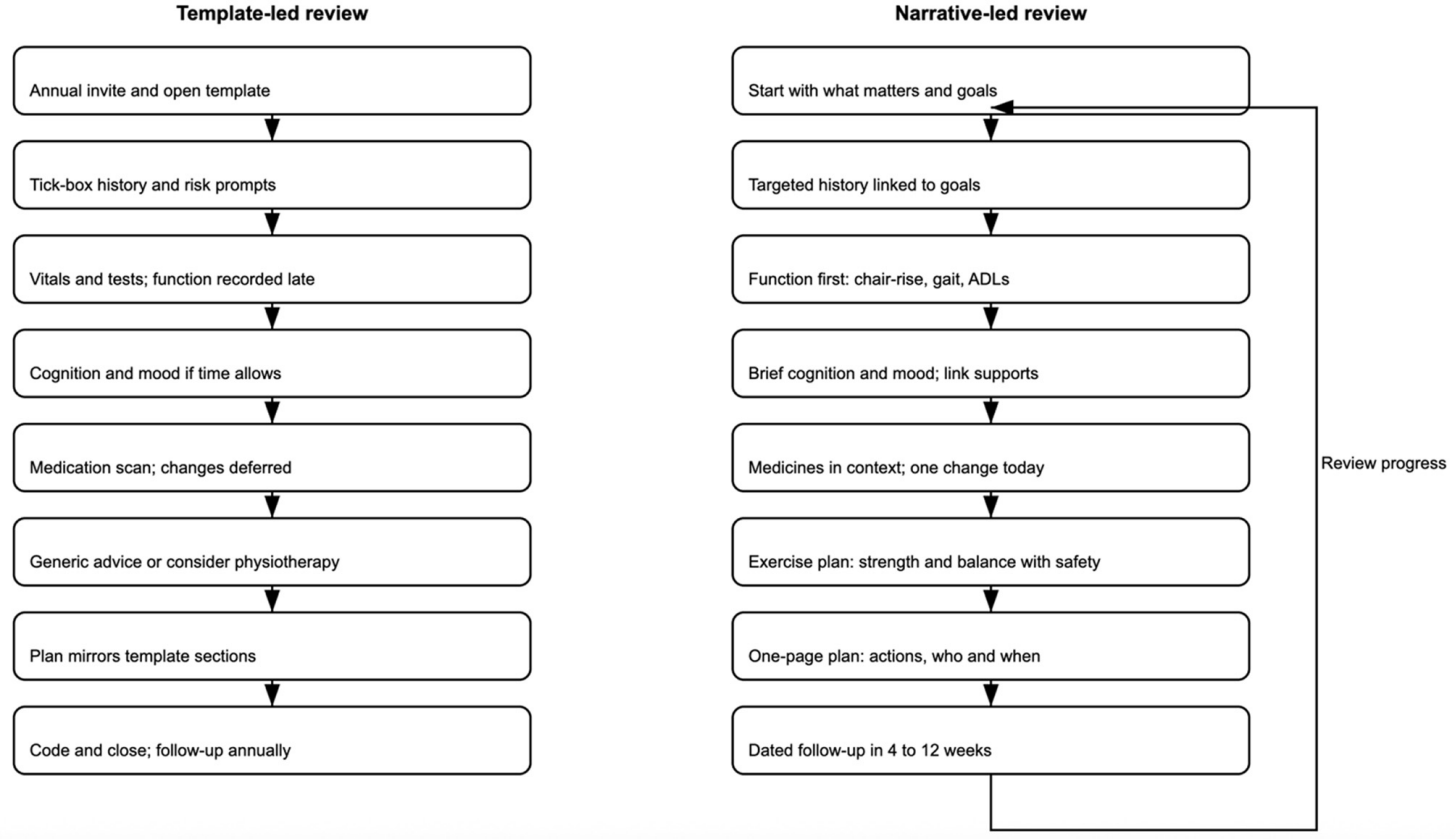
Comparative flows for frailty reviews—template-led pathway **(left)** vs. narrative-led pathway **(right)**. The template-led pathway is linear and checklist-driven; the narrative-led pathway begins with “what matters,” links actions to goals, embeds an exercise prescription, and includes a follow-up feedback loop.

One of the simplest but most effective changes is reversing the order of the conversation itself. Too often, “what matters to me” appears after the discussion of falls, blood pressure, and medication—if it appears at all. Added after such items, the question risks seeming perfunctory or redundant. When placed first, it immediately makes the patient's voice not peripheral, but central. It also makes it easier for clinical information to be reinterpreted in the context of the patient's goals. For instance, dizziness may mean one thing for someone whose chief aim is to keep dancing and another for someone who values getting around the house on stairs. Presenting the review this way invites a more logical and empathic conversation flow. Beginning with goals allows clinical information to be interpreted through that lens. Table 1 illustrates a conceptual comparison between current template-led reviews and a reimagined, narrative-led model, highlighting key differences in structure, clinician role, and patient experience. Figure 1 provides a side-by-side flow of both pathways at a glance.

**Table 1:** Contrasting flows: template-led vs. narrative-led frailty reviews.

**Stage**	**Template-led review (typical flow)**	**Narrative-led review (proposed flow)**
Entry trigger	Annual call/contractual requirement	Patient goal or recent change prompts review
Opening move	Identity checks → template opened	“What matters most right now?” establishes agenda
Information gathering	Tick-box history; generic risk prompts	Targeted history tied to stated goals
Functional assessment	Timed Up & Go/PRISMA etc., recorded late	Simple function first (chair-rise, gait, ADLs) to anchor plan
Cognitive/psychological	Screeners if time allows	Brief cognition/mood screen early; link to supports
Social context	Recorded as living alone/with; little depth	Routines, carers, community assets captured in 2–3 lines
Medication review	Polypharmacy/contraindication scan; changes deferred	Medicines discussed in context of goals; one change agreed today
Risk screening	Falls, pressure sores, nutrition tick boxes	Prioritized risks that block goals; actions assigned
Exercise prescription	General leaflet or “consider physio”	Individualized plan (strength/balance), dosage + safety noted
Care planning	Plan mirror template sections	One-page plan: goal, actions, who/when, review date
Follow-up	Annual or unscheduled	Dated review in 4–12 weeks; mode agreed with patient
Roles/team	GP/nurse completes template solo	Shared tasks (pharmacy, physio, link-worker) recorded clearly
Documentation	Long template output; hard to scan	Brief narrative + key data; patient-friendly summary generated
Measures used	Completion rates; QOF-style items	PROMs/PREMs (usefulness, function, confidence) plus safety
Equity and access	Default English, digital-first invites	Multilingual prompts; non-digital paths and outreach noted
Digital support	Auto-populate, many mandatory fields	Fields for narrative, goal tracking, and quick follow-ups
Patient experience	Process feels impersonal; unclear next steps	Purpose is clear; next step and contact named

Exercise prescriptions must be made explicit in the review. Using the patient's own goals, clinicians should co-create a simple, progressive home- or class-based programme (e.g., chair rises, step-ups, and resistance bands) with clear dosage, safety guidance, and a dated follow-up to review progress. Where appropriate, referral routes (physiotherapy, community strength-and-balance classes) should be offered and recorded alongside the goal.

Continuity of care is another pillar that has been undermined in recent years, particularly in rota-based triage and turnover-driven systems. For frail patients, however, continuity is not a luxury—it is often the means of creation of trust, disclosure, and individualized planning. Seeing the same clinician for serial reviews encourages safety in the relationship. It reduces the need for patients to rehearse sensitive or complex histories, and it allows clinicians to follow up on subtle shifts in affect, engagement, or physical status over time. Continuity also enables longitudinal tracking of goals: a patient's stated priorities—such as attending a family event or feeling secure in walking—can be explored, not restarted annually.

There is good reason to make goal-tracking integral to the review process. Current systems of recording are adequate for recording QOF targets—blood pressure, changes in medication, referral—but poor at having structured fields for non-clinical or functional goals. Allowing patients to nominate goals for themselves and record them in a visible, revisited section of the clinical record would distinguish them from being anecdotal but actionable. Just as the fall in HbA1c is followed over time, so “to walk to the end of the road without aid” or “to cook twice a week” may be followed, enabled, and cheered on. This mirrors findings from Dutch primary care, where structured tracking of personal goals increases motivation and continuity (16). Such goals may appear humble but usually get to the point: of identity, of independence, of pleasure. Patient-defined functional goals that are recorded, revisited, and progressed become as visible as clinical targets.

To achieve this, clinical and admin staff must possess the ability to recognize emotional need—rather than physiological risk. Depression assessments or cognitive testing may be included in frailty reviews, but is done as a binary exercise, not a relational one. Staff training may enhance confidence in handling revelations of loss, loneliness, or fear—not with referral scripts, but by listening and being present. Reception and support staff must also be included, as they are often gatekeepers to the review process and may first identify disengagement, frustration, or confusion—signs that frailty is not just clinical but emotional. A team that recognizes emotional needs as well as physiological risk and aligns process with patient experience.

To develop reviews that are both structured and sensitive, co-production with older people must move from recommendation to routine. Experience-based Co-design (EBCD), now used in the UK, Australia, and Canada, has shown promise in health service improvement by putting the lived experience of service users at its heart and using this to guide concrete system changes (1, 5, 6). In frailty care, co-design workshops can show where standard reviews go wrong: confusing jargon, impenetrable digital portals, and rigid formats that do not reflect changing conditions. Co-design not only improves usability—similar programmes in Europe (18) and New Zealand (23) have shown how older adults' direct input can shift service tone, access, and relevance. When older people are engaged in designing their own care pathways, review is no longer something done to them—and becomes something done with them. Co-design with older adults produces reviews that are usable, humane, and relevant.

Another factor all too often overlooked is follow-up. Frailty reviews, by definition, are annual. The priorities—and the needs—of patients rarely have a 12-month rhythm to them. Systems must facilitate review touchpoints that are aligned with substantive change, whether in health or in the circumstances of life. This does not necessarily involve full re-review; sometimes periodic check-ins to review pre-agreed goals or to acknowledge changes in circumstance may sustain momentum and relationship confidence. Systems might automate such reviews based on clinical data; equally, patients and carers must have open pathways to request early reviews where needed. Follow-up that varies with changes in goals and circumstances is more responsive than a fixed annual rhythm.

It is also time to inquire about what are the measures used to ascertain the quality of a frailty review. Audits now focus on process completion: did the review get coded, were the primary domains covered, did the medication get reconciled? But not many systems capture the depth, relevance, or emotional impact of the interaction. Despite the widespread global adoption of digital templates, few include fields for narrative input, emotional content, or patient priorities beyond care escalation. Did the patient get heard? Did the discussion lead to meaningful action? Was the patient's priority heard and addressed? These are tough to quantify, but not impossible. Patient-reported outcome and experience measures (PROMs and PREMs) in frailty care provide richer information about what happened and how it was experienced. Including PROMs and PREMs captures perceived usefulness and impact, not only completion.

Finally, digital systems must be adapted—not merely to record the review, but to fit its ethos. Templates must invite open questions without restricting responses to pull-down menus. Interface design must accommodate narratives, for goal recording, for collaborative decision-making comments. Multilingual interfaces are also vital in multicultural health systems where older adults may express needs in their first language (1, 5, 6). Where there is digital exclusion, there must be clear alternatives—a concern echoed in Nordic health systems, where aging populations often face similar barriers to access (24). Frailty is multi-dimensional; the systems that support its review must be adapted to the same flexibility. Templates that invite narrative, multilingual input, and alternatives for those digitally excluded better reflect the ethos of the review.

In reworking the frailty review, the intention is not to abandon its structure. The review may and should include clinical assessments, interventions to reduce risk, and medication reviews, but these must be placed within the context of the person, not just the sum of the things that might make them frail. When meaning is accorded the same priority as numbers, the review ceases to be a task to complete. It becomes a relationship. For individuals who live with frailty, it may be the most therapeutic intervention of all.

## Discussion

Frailty reviews are widespread in routine practice, yet the personal value of the encounter is often limited. For older people, the experience is not one of being reviewed but one of being processed—rated, screened, and stratified with little sense of continuity or care. When personalization is mediated by the template, people risk being reduced to data points in systems designed for documentation rather than a relationship (25, 26).

We must be ready to pose tough questions. Not whether the frailty assessment is being conducted, but whether it is being perceived as helpful. Not whether it detects risks, but whether it detects the person. The World Health Organization has called for health systems worldwide to shift from disease-focused models to capacity-enhancing care for older persons, of which frailty review is a key component (27). For patients whose lives are not characterized by admissions but by incremental, subtle loss—mobility, confidence, roles—what matters cannot be elicited from a tick box. These losses are not dramatic, but they are defining (20, 28).

The strongest reversible component of frailty is low strength and function; personalized resistance training improves gait speed, chair-rise time, and independence when reviewed and progressed over time. Embedding this into the frailty review—rather than treating it as optional advice—aligns the review with measurable functional gains that patients' value.

Primary care, long the stronghold of continuity and trust, is where the potential for relationship frailty care still resides. General practice, in contrast with the models of episodic care, has the potential for knowing the patient over years—not as a fall risk or eFI score, but as having fears, relationships, hopes, and a life story. Even there, system design intrudes: rota staffing, rushed appointments, and fractured follow-up threaten to render the review more like a requirement than relationship (29, 30). Similar concerns have emerged in US, New Zealand, and South Korean primary care, where team-based models risk diluting long-term relationships (23, 31, 32).

The danger is not just underperformance—it is misrecognition. Deficit listings without questioning may make patients feel flat, invisible, or—across cultures where independence is prized—experience the loss as existentially disorienting. Emerging research into frailty and identity confirms the importance of form and wording: the way clinicians ask, document, and follow up affects the way patients experience their own aging (19, 33). If we ask just about falls, meds, or mood, we miss the bigger story—what the patient continues to care about, fears losing, or wants to recover.

That is where there needs to be a reframing. Reviewing should not be a static audit but a co-authored one. Frailty, after all, is not just about decline—it is about adaptation, priorities, and how individuals live with uncertainty. Clinical success, in this case, is not always improvement—it could be stability, dignity, or simply being able to fulfill one's promise: to make it to the family reunion, to be able to cook for oneself, or to take the morning walk unassisted (11).

Emerging practice models such as Experience-based Co-design (EBCD) offer practical means to return the review to a relational process (34). Such approaches illustrate that patients do not simply want to be consulted—they want to be involved in co-designing the way care is delivered. When older adults help to design reviews, the questions change, the tone is less adversarial, and the order is more personalized. Measures still matter, but they are placed within a larger narrative that patients recognize as their own.

Organizational direction is starting to recognize this shift. NHS England has consistently demanded the incorporation of personalized care planning within frailty pathways (35). The British Geriatrics Society and NICE have reaffirmed the need for shared decision-making, continuity, and goal-setting in older age care (36, 37). Implementation, however, lags behind intention. Too often, such ideals are aspirational addenda to a template that remains risk-first, patient-second.

Innovation in the care of frailty will not come from more forms or better software. It will come from what happens between people in moments that are all too easy to miss—how a clinician starts an assessment, how they deal with sorrow, and how they work with ambiguity. These are not system inefficiencies; they are the work of care itself. They require time, trust, and education, not protocol (2, 38).

The review, reimagined, could then be another thing entirely: a formal act of recognition. Somewhere the patient's voice controls the tone, where goals, humble though they may be, are documented and spoken to, and where continuity of relationship is not solely for safety but for meaning. If the system will accept this—accept friction, conversation, and slowness—the frailty reviews could serve as a constructive interface between patients and services. Because the question is not whether frailty reviews are being done. It is whether they matter anymore. And to whom?

As frailty frameworks shift globally—from risk reduction to value restoration—frailty reviews must follow suit. This paper argues for a new global language of review: one that captures not just what is lost, but what remains important. A review that starts with goals, embeds continuity, and exercise prescription, tracks what matters, and includes measures of perceived usefulness as well as completion is more likely to be experienced as meaningful.
